# Tumor-associated macrophages derived CCL18 promotes metastasis in squamous cell carcinoma of the head and neck

**DOI:** 10.1186/s12935-018-0620-1

**Published:** 2018-08-28

**Authors:** Li She, Yuexiang Qin, Juncheng Wang, Chao Liu, Gangcai Zhu, Guo Li, Ming Wei, Changhan Chen, Guancheng Liu, Diekuo Zhang, Xiyu Chen, Yunyun Wang, Yuanzheng Qiu, Yongquan Tian, Xin Zhang, Yong Liu, Donghai Huang

**Affiliations:** 1 Department of Otolaryngology Head and Neck Surgery, Xiangya Hospital, Central South University, 87 Xiangya Road, Changsha, 410008 Hunan People’s Republic of China; 2 Otolaryngology Major Disease Research Key Laboratory of Hunan Province, 87 Xiangya Road, Changsha, 410008 Hunan People’s Republic of China; 3 Department of Otolaryngology Head and Neck Surgery, The Second Xiangya Hospital, Central South University, 139 Renmin Road, Changsha, 410010 Hunan People’s Republic of China

**Keywords:** Squamous cell carcinoma of head and neck, Tumor-associated macrophage, CCL18, Metastasis, Epithelial–mesenchymal transition, Stemness

## Abstract

**Background:**

Alternatively activated macrophages in tumor microenvironment is defined as M2 tumor-associated macrophages (M2 TAMs) that promote cancer progression. However, communicative mechanisms between M2 TAMs and cancer cells in squamous cell carcinoma of head and neck (SCCHN) remain largely unknown.

**Methods:**

Quantitative real-time PCR, western blotting, enzyme-linked immunosorbent assay and flow cytometry were applied to quantify mRNA and protein expression of genes related to M2 TAMs, epithelial–mesenchymal transition (EMT) and stemness. Wounding-healing and Transwell invasion assays were performed to detect the invasion and migration. Sphere formation assay was used to detect the stemness of SCCHN cells. RNA-sequencing and following bioinformatics analysis were used to determine the alterations of transcriptome.

**Results:**

THP-1 monocytes were successfully polarized into M2-like TAMs, which was manifested by increased mRNA and protein expression of CCL18, IL-10 and CD206. Conditioned medium from M2-like TAMs promoted the migration and invasion of SCCHN cells, which was accompanied by the occurrence of EMT and enhanced stemness. Importantly, CCL18 neutralizing antibody partially abrogated these effects that caused by conditional medium from M2-like TAMs. In addition, recombinant human CCL18 (rhCCL18) correspondingly promoted the malignant biological behaviors of SCCHN in vitro. Finally, RNA-sequencing analysis identified 331 up-regulated and 363 down-regulated genes stimulated by rhCCL18, which were statistically enriched in 10 cancer associated signaling pathways.

**Conclusion:**

These findings indicate that CCL18 derived from M2-like TAMs promotes metastasis via inducing EMT and cancer stemness in SCCHN in vitro.

**Electronic supplementary material:**

The online version of this article (10.1186/s12935-018-0620-1) contains supplementary material, which is available to authorized users.

## Background

Squamous cell carcinoma of head and neck (SCCHN) originates from the nasal cavities, paranasal sinuses, oral cavity, nasopharynx, oropharynx, hypopharynx and larynx. It ranks as the sixth aggressive malignancy in the world. Although multiple therapies including surgery, radiotherapy and chemotherapy have been applied in clinical settings for decades, the prognosis of SCCHN patients are still unsatisfactory [1]. Lymph node metastasis is one of major causes for a poor prognosis in SCCHN patients [2, 3]. Therefore, it is urgent and imperative to clarify the mechanisms underlying metastasis, which will benefit future surveillance and target therapy for SCCHN patients.

Tumor microenvironment (TME) is composed by diverse cell types including cancer cells, immune cells and multiple molecules such as cytokines, chemokines and metabolites etc. [4, 5]. Communications between cancer cells and stromal cells is important in cancer malignant transformation and progression [6]. Peripheral monocytes infiltrate into tumors, are educated by TME and polarize into classically activated type 1 (M1) macrophages or alternatively activated type 2 (M2) macrophages. Tumor-associated macrophages (TAMs) include M1 and M2 TAMs, M2 TAMs always facilitate angiogenesis, matrix breakdown and cancer cell movement, all of which are indispensable elements for cancer metastasis [7, 8]. Therefore, macrophages represent a valuable therapeutic target for cancer [9–11]. Importantly, clinical data from patient samples indicate that macrophage infiltration is negatively correlated with the poor prognosis in diverse cancers [12–16].

Cancer cells and TAMs have their own specific and/or common secretions and metabolites, which together reprogram each side. For example, TAMs can provide pleiotrophin for glioblastoma to maintain stemness and promote malignant growth [17]. Cancer cells secrete VEGF or CCL2 to recruit monocyte to tumors [18, 19]. In another aspect, cancer cells and TAMs compete essential nutrition for survival, and lactic acid produced by cancer cells also induces polarization of M2 TAMs [20]. However, signals involved in the mutual communication between TAMs and cancer cells are complex and poorly understood.

Chemokine (C-C motif) ligand 18 (CCL18), a member of CC chemokine family, is predominantly produced by M2 TAMs [21]. CCL18 overexpression is observed in breast cancer [12], ovarian cancer [22], and glioma [23] etc., which predicts a poor prognosis. In vitro and in vivo experiments indicate that M2 TAMs-derived CCL18 promotes the metastasis in breast carcinoma [12] and pancreatic ductal adenocarcinoma [15]. However, the effects of CCL18 in SCCHN are poorly unknown [24]. In this study, we confirmed the crosstalk between TAMs and SCCHN cells. Moreover, TAMs derived CCL18 promoted the migration and invasion via inducing epithelial–mesenchymal transition (EMT) and maintaining stemness of SCCHN in vitro.

## Materials and methods

### Cell cultures, treatment and conditional medium collection

SCCHN Tu686 cell line, established from a primary tongue tumor, was kindly provided by Dr. Zhuo Chen (Emory University Winship Cancer Institute, Atlanta, Georgia, USA). SCCHN FaDu cell line, established from a hypopharyngeal tumor removed from a Hindu patient, was purchased American Type Culture Collection (ATCC, VA, USA). Human THP-1 monocytes were obtained from American Type Culture Collection (ATCC, VA, USA). Tu686 cells were cultured in DMEM/F12 medium, FaDu cells were cultured in DMEM medium and THP-1 cells were maintained in RPMI 1640 medium at 37 °C in 5% CO_2_ atmosphere, which containing 10% foetal bovine serum (FBS, Gibco), 100 IU/mL penicillin and 100 μg/mL streptomycin (Gibco).

THP-1 cells were differentiated into M0 macrophages by incubation with 200 ng/mL phorbol 12-myristate 13-acetate (PMA, Sigma, MO, USA) in serum-free medium for 24 h. Then, M0 macrophages were polarized in M2-like TAMs by incubation with 20 ng/mL of recombinant human interleukin 4 protein (rhIL-4, R&D Systems Inc., MN, USA) and 20 ng/mL of recombinant human interleukin 13 protein (rhIL-13, R&D Systems Inc., MN, USA) for another 24 h [25]. The supernatants from M2-like TAMs and M0 macrophages were filtered and defined as M2-like TAMs conditioned medium (CM) and M0 CM, which were used by the addition of 30% fresh complete medium in the following experiments. Tu686 and FaDu cells were incubated with recombinant human CCL18 (rhCCL18) protein (Abnova, CA, USA) at the concentration of 20 ng/mL for 48 h and then subjected to the following experiments.

### Quantitative reverse transcription-polymerase chain reaction analysis (qRT-PCR)

Total RNA was isolated from cells (Tu686, FaDu or monocyte/macrophages) using TRIzol reagent (Invitrogen, CA, USA) according to the manufacturer’s instructions. To quantify the expressions of mRNAs, High Capacity RNA-to-cDNA kit and SYBR^®^ Green PCR Master Mix (Applied Biosystems, CA, USA) were used for reverse transcription and PCR amplification. GAPDH was used as an endogenous control. The expression levels were quantified using the methods of 2^−∆∆Ct^ [26]. All primer sequences were listed in Table 1. Each experiment was performed in triplicate.

**Table 1:** qPCR primers used in this paper

qPCR primers		
Genes	Primer-forward	Primer-reverse
*CD206*	CAG GTG TGG GCT CAG GTA GT	TGT GGT GAG CTG AAA GGT GA
*CCL18*	CTC TGC TGC CTC GTC TAT ACC T	CTT GGT TAG GAG GAT GAC ACC T
*IL*-*10*	GGT TGC CAA GCC TTG TCT GA	AGG GAG TTC ACA TGC GCC T
*CCL22*	ATG GCT CGC CTA CAG ACT GCA CTC	CAC GGC AGC AGA CGC TGT CTT CCA
*CD133*	CAG AGT ACA ACG CCA AAC CA	AAA TCA CGA TGA GGG TCA GC
*CD44*	TGC CGC TTT GCA GGT GTA T	GGC CTC CGT CCG AGA GA
*ALDH1*	TCC TGG TTA TGG GCC TAC AG	CTG GCC CTG GTG GTA GAA TA
*E*-*cadherin*	GCT GGA CCG AGA GAG TTT CC	CAA AAT CCA AGC CCG TGG TG
*Vimentin*	TGT CCA AAT CGA TGT GGA TGT TTC	TTG TAC CAT TCT TCT GCC TCC TG
*Snail*	CCT CCC TGT CAG ATG AGG AC	CCA GGC TGA GGT ATT CCT TG
*Slug*	GCT CAG AAA GCC CCA TTA GTG ATG	GCC AGC CCA GAA AAA GTT GAA TAG
*GAPDH*	TCC AAA ATC AAG TGG GGC GA	AGT AGA GGC AGG GAT GAT GT

### Flow cytometry analysis

To evaluate the expression of surface proteins on macrophages including CD206 and CD163, polarized and nonpolarized macrophages were collected and resuspended in cold PBS containing 2% BSA (Sigma, MO, USA), then incubated with FITC mouse anti-human CD206 (BD Biosciences, CA, USA) and PE mouse anti-human CD163 (BioLegend, CA, USA) on ice for 30 min. To further confirm the stemness of SCCHN cells, Tu686 cells were dissociated into single-cell suspension and resuspended in cold PBS containing 2% BSA (Sigma, MO, USA), then stained with PE-Cy5 anti-human CD133 antibody (BioLegend, CA, USA) and FITC mouse anti-human CD44 antibody (BioLegend, CA, USA) on ice for 1 h. Cells were analyzed by flow cytometry with a FACS Calibur instrument (BD Biosciences, CA, USA). All data was analyzed using FlowJo software (Tree Star Inc., OR, USA). The experiments were performed in twice.

### Enzyme-linked immunosorbent assay (ELISA)

Cytokine levels of CCL18 and IL-10 in supernatants of M0 or M2-like macrophages were determined by a human CCL18/PARC ELISA kit (R&D Systems Inc., MN, USA) and human IL-10 ELISA kit (R&D Systems Inc., MN, USA) according to manufacturer’s instructions. Each experiment was performed in triplicate.

### Wounding-healing and Transwell invasion assay

The wound-healing and invasion assays have been described previously [27–29]. The experiments were performed in triplicate. For wound-healing assay, Tu686 and FaDu cells were seeded into 6-well plates and grown to 80–90% confluence. Then, the cells were disrupted with a standard 10 μL sterile micropipette tip and washed with PBS, which were exposed to different conditioned medium for 48 h. Photographs were captured under phase-contrast microscope.

For Transwell invasion assay, transwell chambers with polycarbonate filter were used, which were coated with Matrigel (Corning, NY, USA) at a concentration of 200 μg/mL. Tu686 or FaDu cells were plated in the upper 24-well Transwell chambers with serum-free medium. Meanwhile, conditioned medium was applied to the lower chamber as chemoattractant for 48 h. After removing the cells on the upper surface of the membrane, 4% formaldehyde and hematoxylin and eosin (HE) were used to fix and stain the cells, respectively. Invaded cells were counted and photographed under a microscope.

### Western blotting

Whole cell protein extracts were collected and western blot assays were performed as we previously described [27, 28]. 30–50 μg total protein extracts were separated by 8–12% SDS-PAGE and then transferred onto polyvinylidene difluoride membrane (Millipore, MA, USA). The blotted membranes were incubated with primary antibodies and subsequently incubated with an HRP-labeled secondary antibody. β-actin (1:2000; Beyotime, China) was used as a loading control. All antibodies in our study are listed as followed: N-cadherin (1:1200; Proteintech Group Inc., IL, USA); E-cadherin (1:1200; Proteintech Group Inc., IL, USA); Vimentin (1:1200; Proteintech Group Inc., IL, USA); Snail (1:500; Santa Cruz, TX, USA); Slug (1:500; Santa Cruz, TX, USA); HRP-anti-mouse or anti-rabbit IgGs (1:2000; Beyotime, China). Each experiment was performed in triplicate.

### Sphere culture and formation assay

For tumorsphere formation assay, Tu686 cells were cultured as a monolayer before being harvested as single cell suspension. Cells were suspended in tumorsphere medium (serum-free DMEM/F-12 medium supplemented with 20 ng/mL human recombinant epidermal growth factor (EGF; Millipore, MA, USA), 20 ng/mL human recombinant basic fibroblast growth factor (bFGF; PeproTech, NJ, USA) and 2% B27), and then subsequently plated in ultra-low attachment 24-well plates (Corning, NY, USA) at a density of no more than 2000 cells/well. Sphere formation was allowed for 10–14 days [30, 31]. The experiments were performed in triplicate.

### RNA-sequencing analysis

Total RNA from Tu686 cells with or without rhCCL18 stimulation was extracted and quantified, library preparation and sequencing. The libraries were sequenced on the Illumina Hiseq 4000 platform. Reads containing adapter or poly-N and reads of low quality were removed from raw data to generate clean reads. Based on the clean reads, Q20, Q30, and GC content of the clean data were calculated. Next, mapped reads were obtained through aligning clean reads to the human genome reference (hg19) by TopHat v2.0.9. To determine expression level of each unigenes, the number of mapped clean reads was counted and normalized into reads per kb per million reads (RPKM).

### GO and KEGG enrichment analysis

Gene Ontology (GO) enrichment analysis of differentially expressed genes were implemented by the GOseq R package, in which gene length bias was corrected. GO terms with corrected *P* < 0.05 were considered significantly enriched by differential expressed genes. Kyoto Encyclopedia of Genes and Genomes (KEGG) is a database resource for understanding high-level functions and utilities of the biological system, such as the cell, the organism and the ecosystem, from molecular-level information, especially large-scale molecular datasets generated by genome sequencing and other high-throughput experimental technologies (http://www.genome.jp/kegg/). We used KOBAS software to test the statistical enrichment of differential expression genes (DEGs) in KEGG pathways.

### Statistical analysis

All data were evaluated using IBM SPSS version 13.0. Unpaired *t* test or one-way ANOVA test was performed to analyze the significant differences between groups. The quantitative data in this study were expressed as the mean ± standard deviation (SD). Differences were considered statistically significant at the value of *P *< 0.05.

## Results

### In vitro polarization of THP-1 cells into M2-like TAMs

**Fig. 1:**
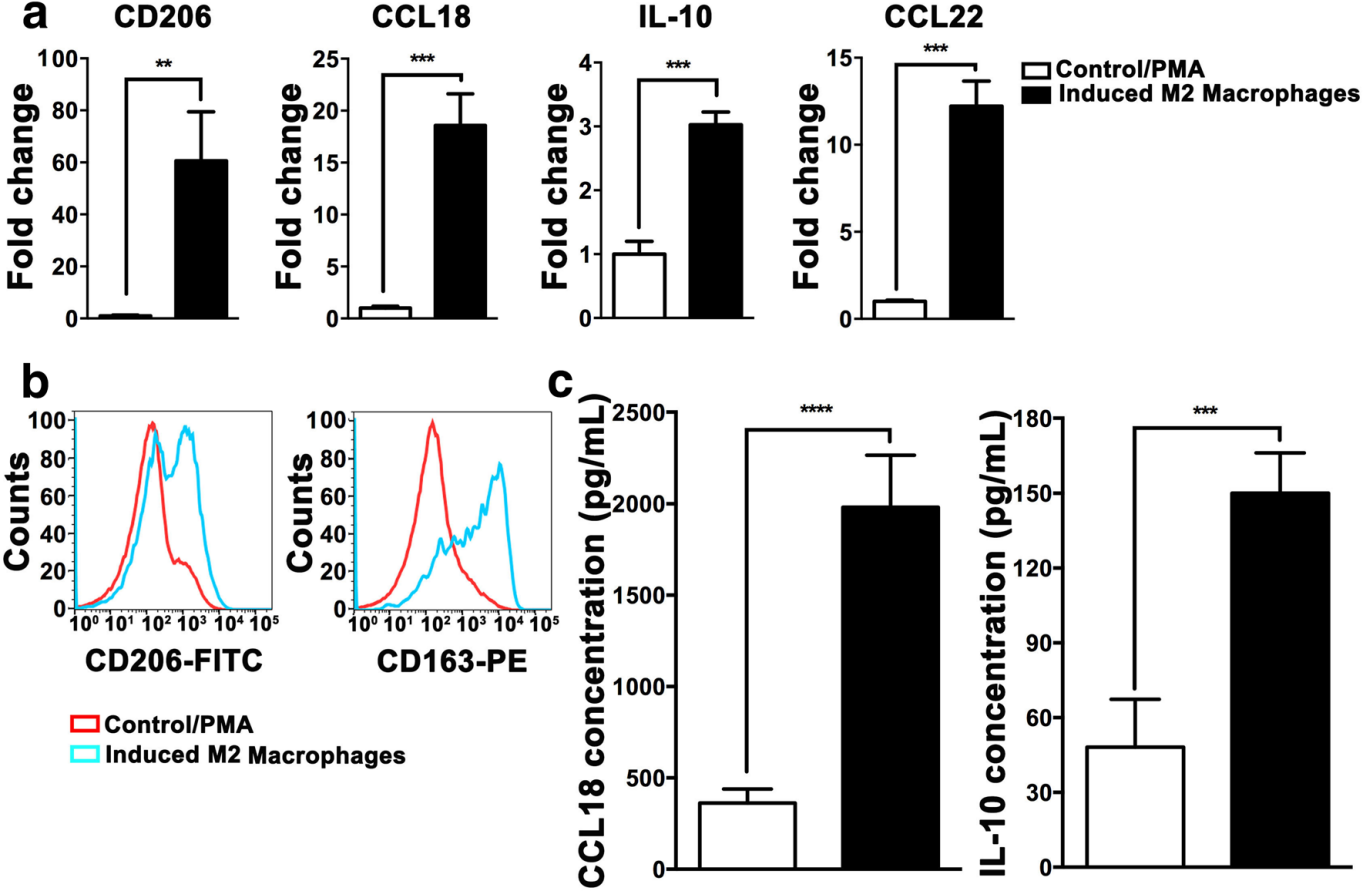
In vitro polarization of THP-1 cells into M2-like TAMs. THP-1 monocytes were treated with the combination of PMA, rhIL-4 and rhIL-13. **a** mRNA expression of M2 macrophages markers (CD206, CCL18, IL-10 and CCL18) was quantified by qRT-PCR. **b** Cell surface proteins of CD206 and CD163 were analyzed by flow cytometry. **c** CCL18 and IL-10 secretion in culture medium was measured by ELISA. Results are shown as mean ± SD. **P <0.01, ***P < 0.001, ****P < 0.0001

Monocytes can be induced into M2-like TAMs via combinational stimulation with PMA, rhIL-4 and rhIL-13 in vitro [25, 32, 33]. Hence, we used this protocol to polarize THP-1 monocytes. M2-like TAMs is characterized by high expression of scavenging receptor CD163, mannose receptor CD206 and increased secretion of cytokines such as IL-10, CCL18 and CCL22 etc. Upon 24 h stimulation, qPCR data clearly showed that cytokine mRNAs including IL-10, CCL18 and CCL22 were dramatically increased (Fig. 1a). ELISA assays further confirmed that IL-10 and CCL18 proteins were correspondingly elevated in the culture medium from polarized macrophages (Fig. 1c). FACS analyses revealed that expression of M2 macrophage membrane receptors including CD163 and CD206 was increased (Fig. 1b). These data indicate that THP-1 monocytes are successfully polarized into M2-like TAMs in vitro.

### M2-like TAMs promote migration and invasion of SCCHN cells in vitro

**Fig. 2:**
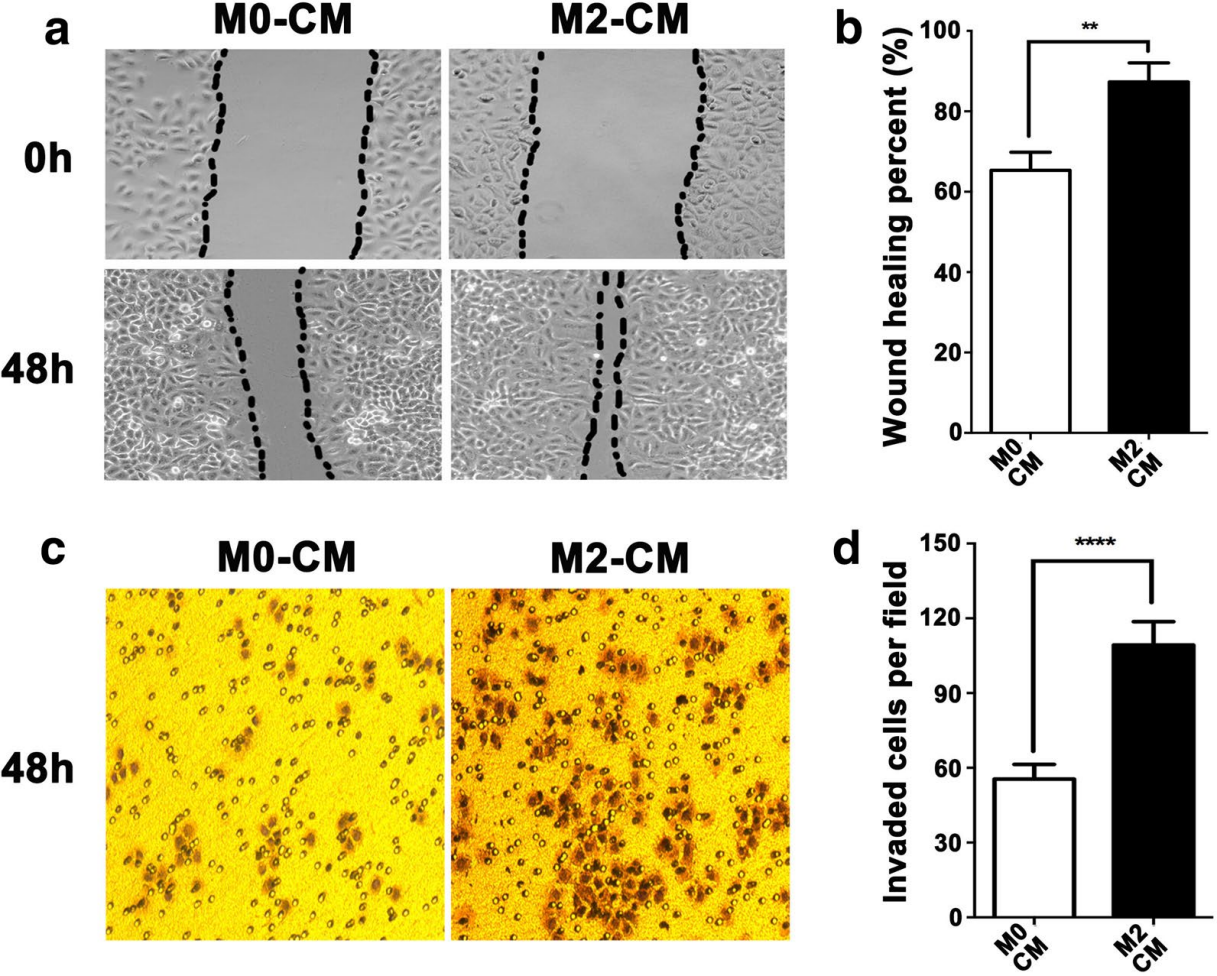
M2-like TAMs promote migration and invasion of SCCHN in vitro. Tu686 cells cultured with M2 CM or M0 CM for 2 days. Wounding-healing assays (**a**, **b**) and Transwell chamber assays (**c**, **d**) were used to measure the changes in migration and invasion of Tu686 cells. Data are shown as mean ± SD. **P <0.01, ****P < 0.0001

To confirm potential communications between M2-like TAMs and SCCHN cells, conditional medium (CM) from unpolarized (M0 CM) and polarized M2-like TAMs (M2 CM) were collected and used in the following experiments. Our data clearly showed that wound-healing ability of Tu686 cells cultured with M2 CM significantly enhanced at 48 h compared with cells treated with M0 CM (Fig. 2a, b). Consistent with wound healing results, Transwell chamber assays revealed that M2 CM promoted the invasive capacity of Tu686 cells (Fig. 2c, d). In addition, M2 CM treatment showed only mild but not statistically different influence on the cell proliferation in vitro. These data indicate that M2-like TAMs enhance the migration and invasion of SCCHN cells in vitro.

### M2-like TAMs induce EMT and stemness in SCCHN in vitro

**Fig. 3:**
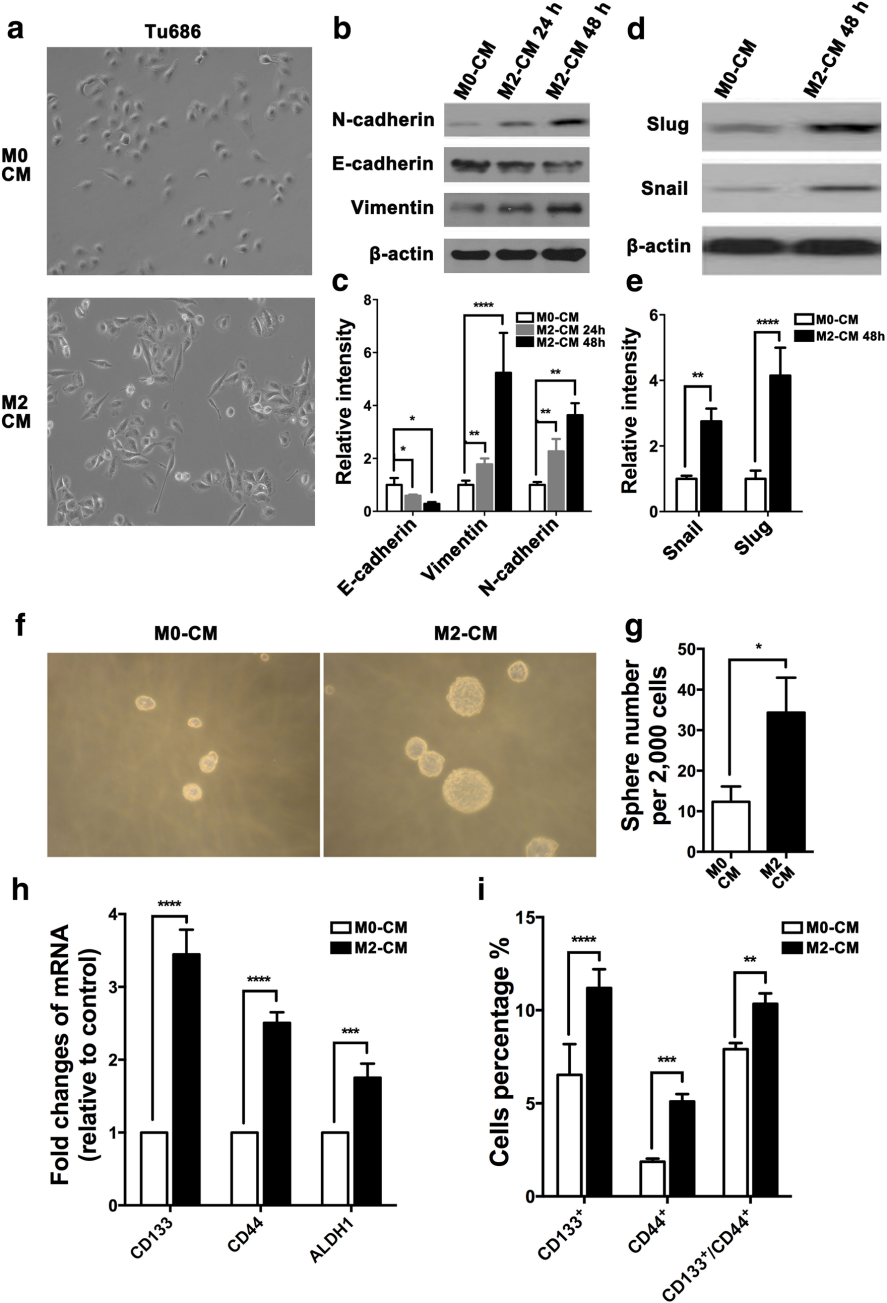
M2-like TAMs induce EMT and stemness in SCCHN in vitro. Tu686 cells cultured with M2 CM or M0 CM for 2 days. **a** Representative morphological images (original magnification 100×). **b**, **c** EMT proteins were examined and semi-quantified by western blotting. Similar with **b** and **c**, proteins (**d** and **e**) of EMT transcription factors Slug and Snail were also measured. **f**, **g** Images of tumorspheres and their quantification were shown. **h** mRNA expression of CSCs markers CD133, CCL44 and ALDH1 was quantified by qPCR. **i** Proteins of CD133 or/and CD44 were analyzed by flow cytometry. Results are shown as mean ± SD. *P < 0.05, **P <0.01, ***P < 0.001, ****P < 0.0001

Epithelial–mesenchymal transition (EMT) is tightly associated with cancer metastasis, which is characterized by canonical morphological and molecular alterations [28, 29]. Upon M2 CM treatment for 3 days, morphology of Tu686 cells transformed to a fibroblast-like shape with less cell–cell contact (Fig. 3a). Moreover, M2 CM induced gradual downregulation of epithelial marker E-cadherin and upregulation of mesenchymal markers N-cadherin and Vimentin at protein level (Fig. 3b, c). Snail and Slug are two critical transcription factors for EMT. M2 CM also significantly increased the expression of Snail and Slug at protein level in Tu686 cells (Fig. 3d, e).

Increasing evidence has demonstrated that cancer cells undergoing EMT changes always increase their stemness [31]. Therefore, we examined the effects of M2 CM on SCCHN stemness. Sphere formation assays revealed that M2 CM treatment generated more and larger tumorspheres in Tu686 cells in vitro (Fig. 3f, g), indicating that M2 CM enhanced SCCHN stemness. Correspondingly, qPCR data showed that M2 CM significantly increased mRNA expression of SCCHN stem markers including CD133, CD44 and ALDH1 (Fig. 3h). FACS analysis further confirmed that M2 CM increased the proportions of CD133^+^, CD44^+^ and CD133^+^CD44^+^ Tu686 cells (Fig. 3i). Taken together, the above data clearly reveal that M2-like TAMs boost EMT and enhance stemness of SCCHN cells, which in turn leads to phenotype changes associated with SCCHN metastasis.

### CCL18 participates in M2-like TAMs mediated metastasis of SCCHN in vitro

**Fig. 4:**
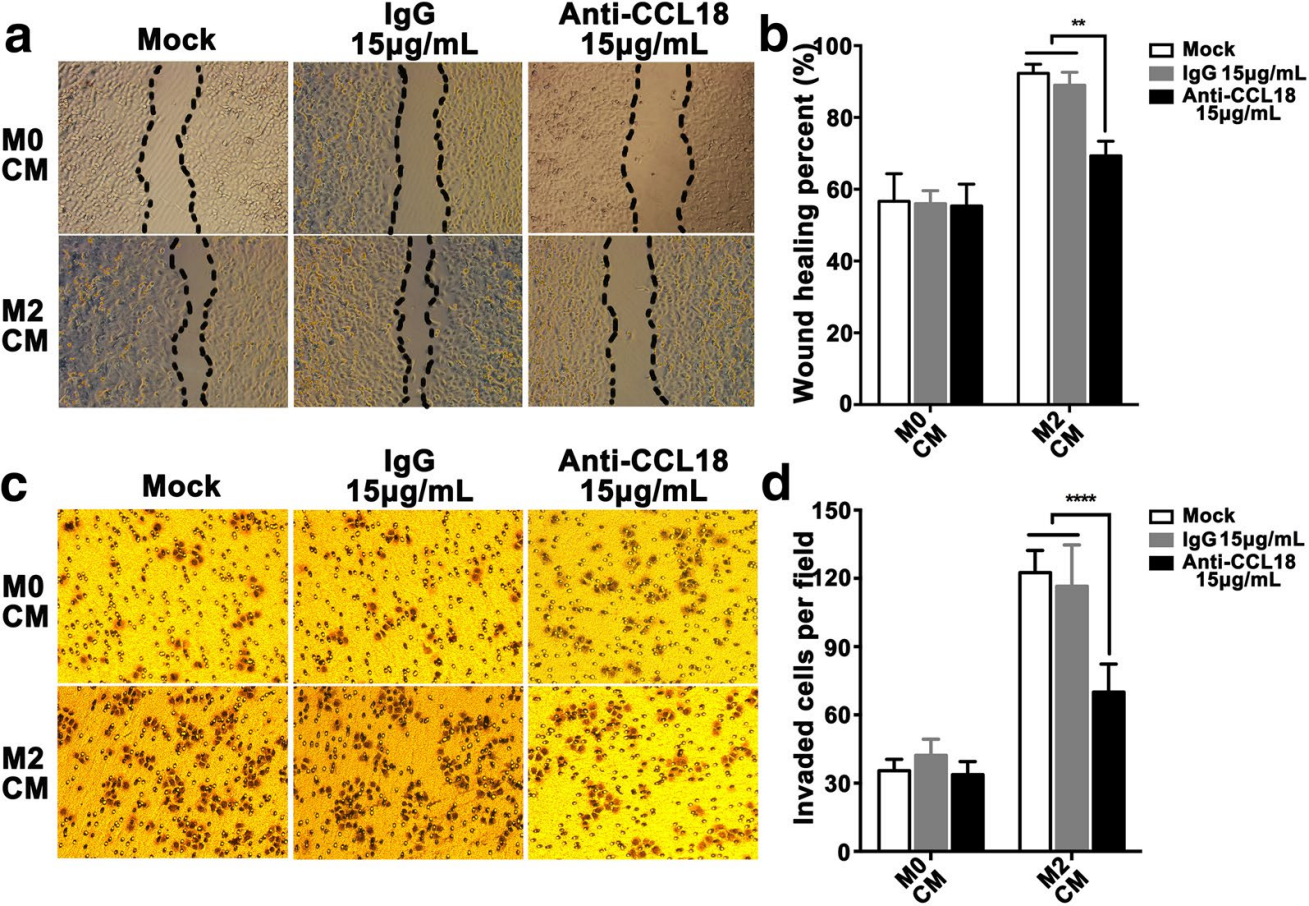
CCL18 participates in M2-like TAMs mediated metastasis of SCCHN in vitro. Tu686 cells cultured with M2 CM or M0 CM in the presence or absence of an anti-CCL18 neutralizing antibody at 15 μg/mL, or an isotype-matched IgG protein. Wounding-healing assays (**a**, **b**) and Transwell chamber assays (**c**, **d**) were used to measure the changes in migration and invasion of Tu686 cells. Data are shown as mean ± SD. **P <0.01, ****P < 0.0001

CCL18, as one of the most important cytokines secreted by M2 TAMs, functions critically in cancer metastasis. As showed in Fig. 1c, CCL18 secretion was drastically elevated in polarized M2-like TAMs. To confirm whether CCL18 directly functioned in M2-like TAMs mediated SCCHN metastasis, specific neutralizing antibody was used to block the binding between CCL18 and its receptor. As anticipated, CCL18 neutralizing antibody at the concentration of 15 μg/mL successfully impeded the migration (Fig. 4a, b) and invasion (Fig. 4c, d) of Tu686 cells enhanced by M2 CM. However, IgG isotype recombinant protein had no reversal effects (Fig. 4a, d). Although CCL18 neutralizing antibody only partially reversed the effects of M2 CM (Fig. 4a, d), these data still demonstrate that CCL18 contributes to enhanced metastasis that induced by M2-like TAMs.

### CCL18 involves in M2-like TAMs induced EMT and stemness in SCCHN in vitro

**Fig. 5:**
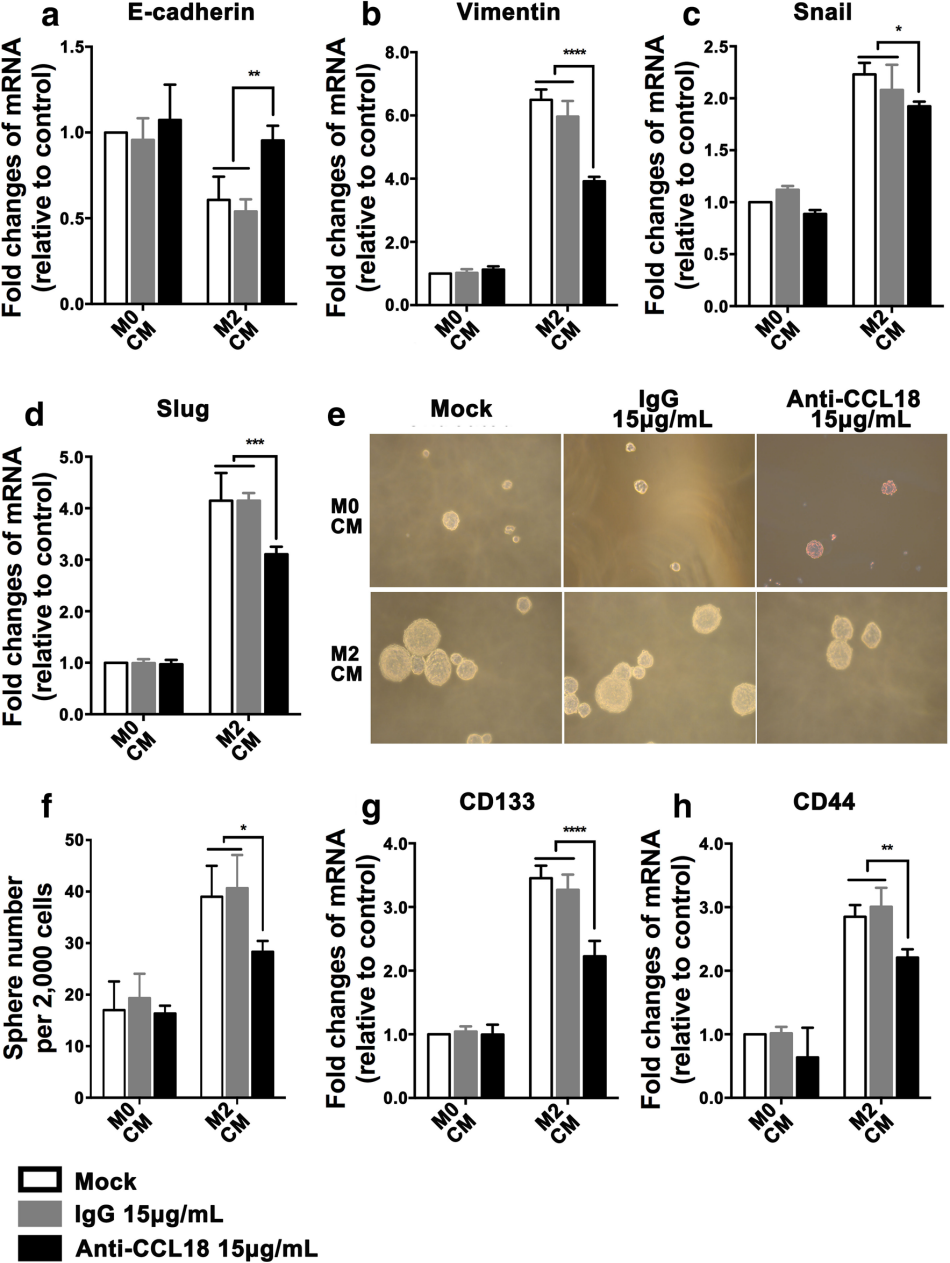
CCL18 involves in M2-like TAMs induced EMT and stemness in SCCHN in vitro. Tu686 cells cultured with M2 CM or M0 CM in the presence or absence of an anti-CCL18 neutralizing antibody at 15 μg/mL, or an isotype-matched IgG protein. **a**–**d** qRT-PCR assays were used to check mRNA expression of EMT markers E-cadherin (**a**), Vimentin (**b**), and EMT transcription factors Snail (**c**) and Slug (**d**). **e**, **f** Representative images for tumorsphere formation and the number of tumorspheres. **g**, **h** mRNA expression of CSCs markers CD133 (**g**) and CD44 (**h**) was quantified by qPCR. Data are shown as mean ± SD. *P < 0.05, **P <0.01, ***P < 0.001, ****P < 0.0001

We also investigated whether CCL18 neutralizing antibody could reverse EMT and stemness mediated by M2 CM. qPCR data indicated that neutralizing antibody completely restored mRNA expression of epithelial marker E-cadherin, partially inhibited the elevation of mesenchymal marker Vimentin, Snail and Slug (Fig. 5a, d). Meantime, size of tumorsphere became smaller and number decreased after the addition of neutralizing antibody (Fig. 5e, f), which was accompanied by declined mRNA expression of stem cell genes including CD133 and CD44 (Fig. 5g, h). All these results suggest that M2-like TAMs-derived CCL18 promotes changes of phenotype associated with SCCHN metastasis via inducing EMT and stemness.

### CCL18-mediated effects on FaDu cells

**Fig. 6:**
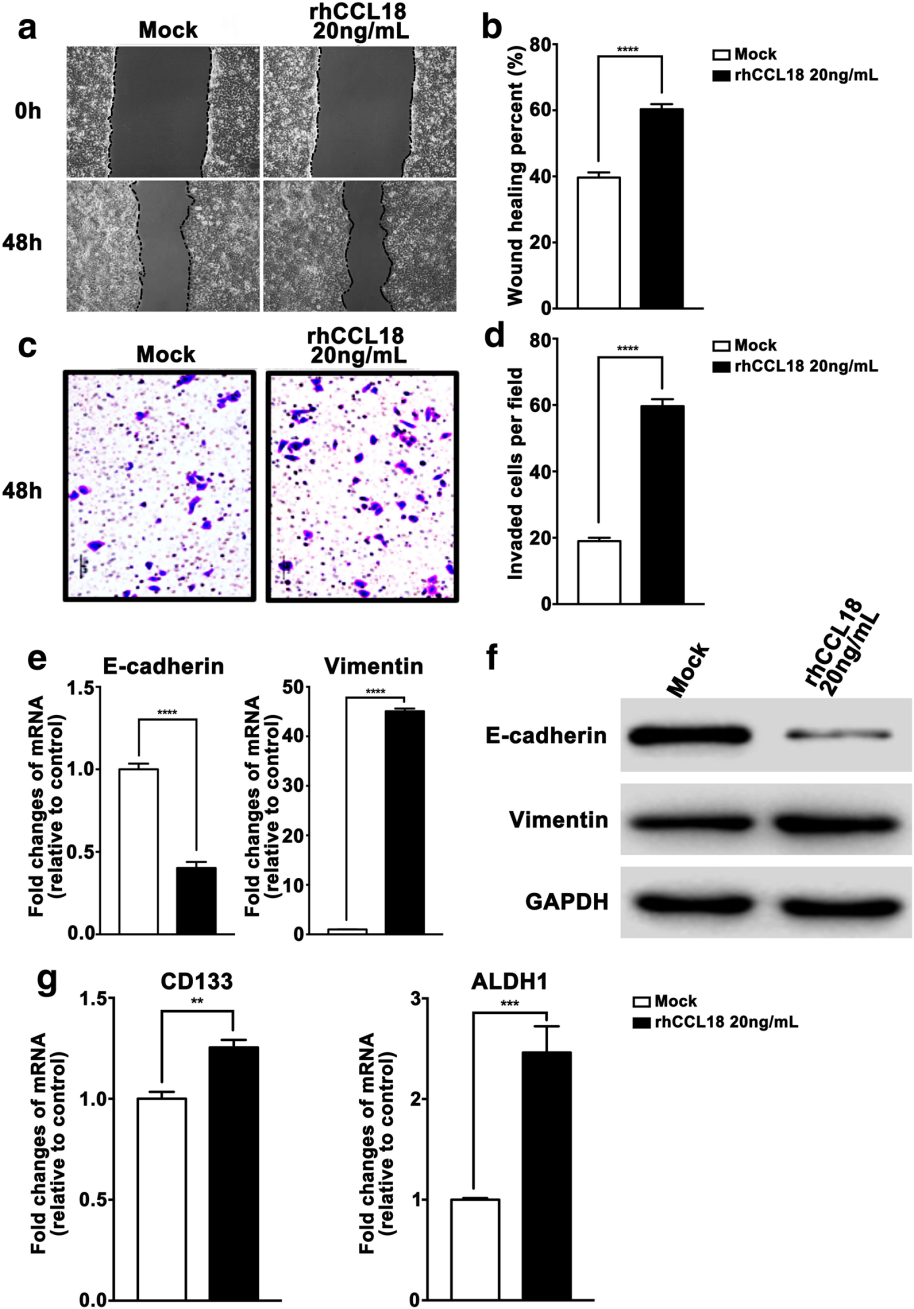
CCL18 mediated effects on FaDu cells. SCCHN FaDu cells were treated by rhCCL18 for 3 days and then subjected to the following assays. **a**, **b** Migratory changes were examined by wound healing assays and quantified. **c**, **d** Invasive ability was assayed by Transwell chamber assays and quantified. **e**, **f** mRNA and proteins associated with EMT were leveled by qPCR and western blotting assays. **g** mRNAs of CSCs markers CD133 and ALDH1 were quantified by qPCR. **P <0.01, ***P < 0.001, ****P < 0.0001

To further strengthen the reliability of our conclusion and to exclude the possibility that the effect of CCL18 on SCCHN was Tu686 cell specific, another SCCHN FaDu cell line was used. As expected, rhCCL18 stimulation obviously enhanced the wound healing (Fig. 6a, b) and invasive ability (Fig. 6c, d) of FaDu cells in vitro. Accordingly, rhCCL18 treatment declined the expression of E-cadherin and increased the expression of Vimentin at both transcriptional and translational levels (Fig. 6e, f). In addition, mRNA expression of CSCs markers CD133 and ALDH1 was also upregulated when exposed to rhCCL18 (Fig. 6g). These findings in FaDu cells are consistent with that in Tu686 cells, which consolidates the conclusion that CCL18 promotes metastasis-associated phenotypes via the induction of EMT and stemness in SCCHN.

### Identification of CCL18-regulated genes and pathways

**Fig. 7:**
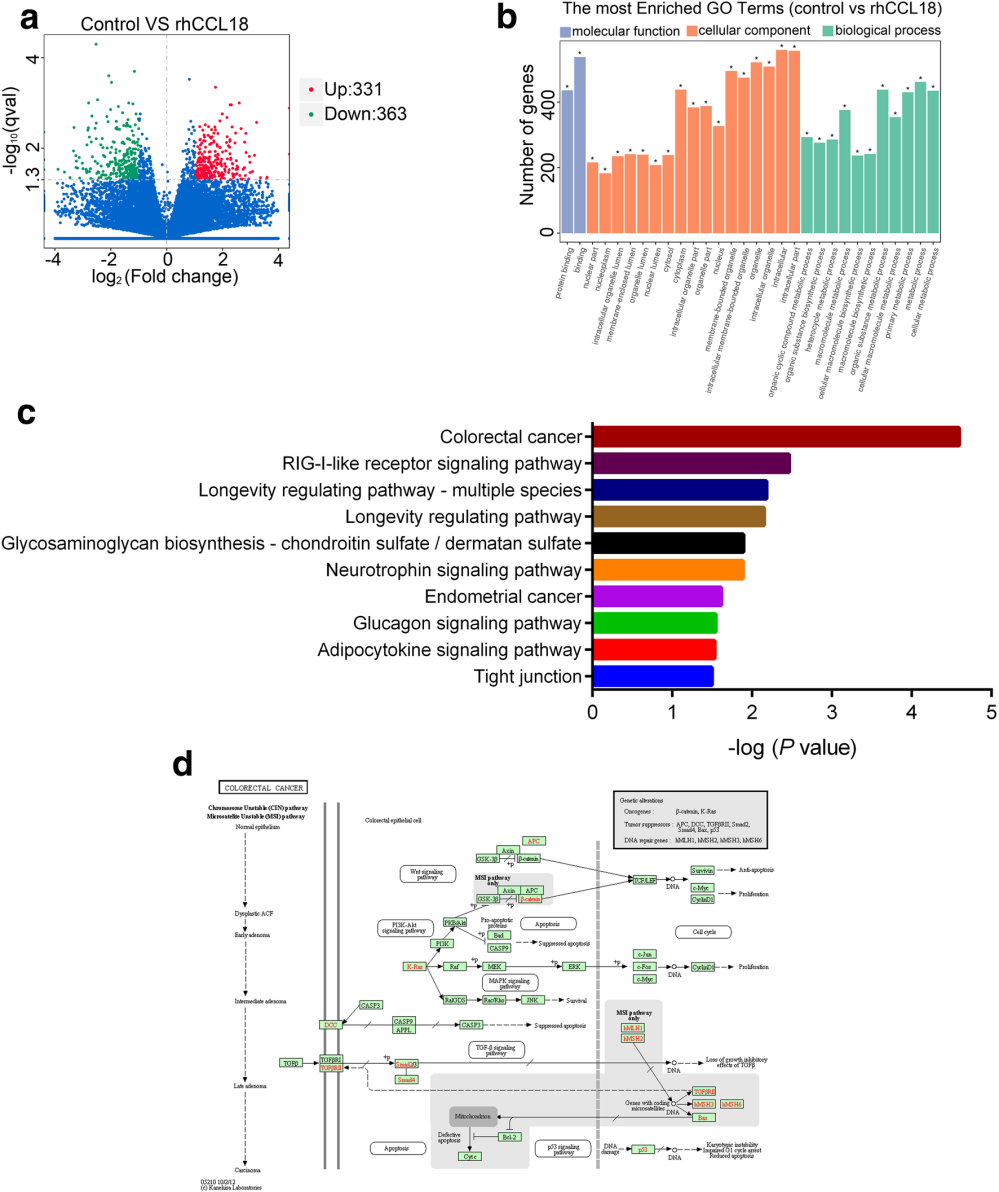
Identification of CCL18-regulated genes and signaling pathways in SCCHN cells. **a** Volcano plot representing the RNA-sequencing results. Red dots indicated up-regulated genes induced by rhCCL18; blue dots were down-regulated genes at a cutoff criteria of fold change ≥ 2.0 and *P* < 0.05. **b** Go analysis including the biological process, molecular function and cellular component were performed on the 694 DEGs. **c** The top 10 signaling pathways analyzed by the KEGG signaling pathway enrichment analysis. **d** KEGG pathway map of the colorectal cancer pathway with DEGs indicated

To better understand the underlying molecular mechanism, Tu686 cells treated with or without rhCCL18 for 3 days were subjected to RNA-sequencing. With the cutoff criteria of an absolute fold-change ≥ 2.0 and *P* < 0.05, a total of 694 genes were differentially expressed, in which 331 genes were increased and 363 genes were declined with CCL18 stimulation (Fig. 7a and Additional file 1). In addition, GO biological process, molecular function and cellular component for the 694 DEGs were also analyzed (Fig. 7b and Additional file 2). KEGG pathway analysis revealed that 10 pathways were significant enriched after rhCCL18 stimulation (*P* < 0.05), which included colorectal cancer and RIG-1-like receptor signaling pathway etc. (Fig. 7c and Additional file 3). The colorectal cancer pathway, as the most significant pathway, contained 12 DEGs (Fig. 7d and Additional file 3).

## Discussion

M2 TAMs, one of the inflammatory immune cells in tumor microenvironment, functions importantly in cancer initiation and progression [34–36]. In our current study, monocyte THP-1 was polarized into M2-like TAMs via the combination stimulation with PMA, IL-4 and IL-13. M2-like TAMs significantly promoted the migration and invasion of SCCHN cells in vitro, which was tightly associated with EMT and stemness. More importantly, we found that CCL18 in M2-like TAMs induced EMT and stemness and then led to SCCHN metastasis. Taken together, our current demonstrated that CCL18 derived from M2-like TAMs may drove SCCHN metastasis via inducing EMT and stemness.

M2 TAMs secret diverse cytokines, enzymes and chemokines, which are involved in cancer malignant progression. CCL16, IL-10, Arginase 1, YM1, VEGF and CCL18 are routine secretions from M2 TAMs under different circumstances [37]. In the secretory profile, VEGF is a well-known pro-angiogenesis protein that facilitates cancer metastasis [38]. IL-10 is a pleiotropic cytokine that is important in inflammation-induced carcinogenesis [39]. Arginase 1 metabolizes l-arginine and participates in the remodeling of T cell functions and tissue repair [40]. These secretions form a complicated network that connects TAMs, tumor cells and other stroma cells in TME. The mutual interactions reprogram each cell type in TME and evolve together with cancer progression. In our current study, M2-like TAMs CM induces EMT, sustains cancer stemness and drastically enhances the capacity of migration and invasion of SCCHN cells, which reveals that specific secretory alteration after M2 polarization of macrophages may contribute to SCCHN metastasis. These data are consistent with previous investigations in other solid tumors [12, 15, 17].

CCL18 is mainly produced by M2 TAMs, which has been reported to promote cancer metastasis via binding to its receptor Nir1 (also named PITPNM3) in multiple human malignancies including breast carcinoma [12], hepatocellular carcinoma [41], ovarian cancer [42], nasopharyngeal carcinoma [43] and lung cancer [44] etc. Therefore, we used neutralizing antibody to block the binding of CCL18 to its receptor Nir1. As anticipated, neutralizing antibody successfully impedes the migration, invasion and markers of EMT and stemness in SCCHN cells in vitro. These data indicate that CCL18 produced by M2 TAMs contributes to the phenotypes associated with metastasis in SCCHN.

However, we have to mention that CCL18 neutralizing antibody only partially inhibits the function of M2-like TAMs, which means that other mechanisms are also involved in mutual communication between TAMs and cancer cells. The phenomenon can be logically explained by the following reasons. Firstly, our current study only focus on cytokine CCL18, it is reasonable to assume that other secretory proteins may also tightly associated with M2 TAMs mediated cancer progression. Secondly, nutrition competition and cell metabolites such as lactic acid and fumarate etc., are critical and indispensable components of CM, which have been intensively investigated and reported to function in cancer progression [20, 45]. Thirdly, the cell–cell communications also exist among other cell types. For instance, M2 TAMs display high expression of PD-L1 that accelerates the exhaustion of tumor infiltrating lymphocytes such as CD8 T cells, leads to suppressive antitumor immunity and cancer progression [46].

CCL18 exerts its function via binding to the receptor Nir1 on cancer cell surface and then activates downstream signaling pathways, which include the mammalian target of rapamycin (mTOR) [47], NF-κB [41] and PI3K/Akt signaling pathways [48] etc. We have found that activation of CCL18/Nir1 signaling pathway can increase the expression of oncogene MTDH, which is important for the promotion of SCCHN metastasis in our previous studies (Data are prepared in another manuscript) [27, 28, 49]. In addition, high throughput sequencing techniques are also employed in our lab to systematically and thoroughly explore the potential downstream signaling components of CCL18/Nir1. Our RNA-sequencing analysis indicates that rhCCL18 induces a transcriptome change that includes 331 up-regulated and 363 down-regulated genes. To thoroughly investigate the molecular mechanism of CCL18, KEGG analysis further demonstrate that 10 signaling pathways are enriched by these 694 DEGs. In these enriched pathways, colorectal cancer signaling pathway, ranking in the first place, includes 12 DEGs caused by CCL18 stimulation, which is involved in cancer initiation and progression. Among other pathways, RIG-I-like receptors signaling pathway is tightly associated with antiviral immunity and inflammatory response [50]. Other pathways did not seem to be directly associated with cancer at first glance. This can be explained by the notion that KEGG analyses are based on available published online data and that these genes involved in the enriched pathways are annotated not only to cancer, but also to many other diseases or biological functions.

## Conclusion

Taken together, our study suggests that M2 TAMs produce CCL18 to induce EMT and cancer stemness, which in turn leads to SCCHN metastasis. However, in-depth molecular mechanism investigation and further in vivo animal models are required to further strengthen our current conclusion.

## Additional files

**Additional file 1. **Differentially expressed genes (DEGs) of Tu686 cells stimulated by rhCCL18

**Additional file 2.** GO Biological Process, Molecular Function, and Cellular Component for the DEGs

**Additional file 3. ** KEGG pathway enrichment analysis for the DEGs

## Abbreviations

TAMs: tumor-associated macrophages
TME: tumor microenvironment
CCL18: Chemokine (C-C motif) ligand 18
EMT: epithelial–mesenchymal transition
SCCHN: squamous cell carcinoma of the head and neck
DEGs: differential expression genes
